# Survey of suspected dysphagia prevalence in home-dwelling older people using the 10-Item Eating Assessment Tool (EAT-10)

**DOI:** 10.1371/journal.pone.0211040

**Published:** 2019-01-23

**Authors:** Kumi Igarashi, Takeshi Kikutani, Fumiyo Tamura

**Affiliations:** 1 Division of Clinical Oral Rehabilitation, The Nippon Dental University Graduate School of Life Dentistry, Tokyo, Japan; 2 The Nippon Dental University, Tama Oral Rehabilitation Clinic, Tokyo, Japan; 3 Division of Rehabilitation for Speech and Swallowing Disorders, The Nippon Dental University, Tokyo, Japan; University of Wisconsin Madison, UNITED STATES

## Abstract

**Objective:**

This study was carried out to determine the prevalence of suspected dysphagia and its features in both independent and dependent older people living at home.

**Materials and methods:**

The 10-Item Eating Assessment Tool (EAT-10) questionnaire was sent to 1,000 independent older people and 2,000 dependent older people living at home in a municipal district of Tokyo, Japan. The participants were selected by stratified randomization according to age and care level. We set the cut-off value of EAT-10 at a score of ≥3. The percentage of participants with an EAT-10 score ≥3 was defined as the prevalence of suspected dysphagia. The chi-square test was used for analyzing prevalence in each group. Analysis of the distribution of EAT-10 scores, and comparisons among items, age groups, and care levels to identify symptom features were performed using the Kruskal-Wallis test and Mann-Whitney U test.

**Results:**

Valid responses were received from 510 independent older people aged 65 years or older (mean age 75.0 ± 7.2) and 886 dependent older people (mean age 82.3 ± 6.7). The prevalences of suspected dysphagia were 25.1% and 53.8%, respectively, and showed significant increases with advancing age and care level. In both groups, many older people assigned high scores to the item about coughing, whereas individuals requiring high-level care assigned higher scores to the items about not only coughing but also swallowing of solids and quality of life.

**Conclusion:**

In independent people, approximately one in four individuals showed suspected dysphagia and coughing was the most perceivable symptom. In dependent people, approximately one in two individuals showed suspected dysphagia and their specifically perceivable symptoms were coughing, difficulties in swallowing solids and psychological burden.

## Introduction

Dysphagia causes dehydration[1] and malnutrition[2] and increases the risk of aspiration pneumonia[3] and mortality[4]. It also impacts quality of life (QOL) [5] and social well-being [6] for the older people. In 2015, the mortality rate from pneumonia was approximately 9.4% [7], and aspiration pneumonia accounted for a majority of older people pneumonia cases in Japan [8]. In order to prevent aspiration pneumonia, it is necessary to establish a social system that enables early detection of and intervention for dysphagia. Thus, it is important to determine the prevalence and features of dysphagia.

Several studies have focused on the prevalence of dysphagia. [9–11] As prevalences are expected to vary substantially depending on the age and the sampling procedure of participants, random sampling should be used for participant selection. Few studies have sought to determine the prevalence of dysphagia in randomly selected community-dwelling individuals [12]. Serra-Prat *et al*. used Volume-Viscosity Swallow Test (V-VST) to detect dysphagia in independent individuals selected by stratified randomization [12]. However, there is no report investigating the prevalence of dysphagia focus on various symptoms in independent and independent older people selected by stratified randomization according to age and care level.

In recent years, the 10-Item Eating Assessment Tool (EAT-10) [13,14], a self-administered questionnaire for dysphagia screening has been widely used. It consists of 10 items, with each item answered on a 5-point scale from 0 to 4 (“no problem” to “severe problem”); higher scores indicate a self-perception of a high level of dysphagia severity. In individuals with >3 points, dysphagia is suspected and referral to a specialist is recommended [13,14].

In this study, the participants were selected by stratified randomization according to age and care level. This study was carried out to determine the prevalence of suspected dysphagia and its features in both independent and dependent older people using EAT-10.

## Methods

This survey was carried out as part of a regional survey on eating/swallowing conducted by a local government. The numbers of independent and dependent older people aged 60 years or older residing in a city in the Tama area of Tokyo, Japan were 47,000 and 7500, respectively. Sample size was calculated to ensure a power = 95% (type II error), and the level of significance at ≤5% (type I error). The effect size was assumed to be medium. The response rate for another survey conducted in this city was 43.54% (data not shown). Therefore, we planned to send the EAT-10 to more than 701 older people in this study. G*Power 3.1.9.2 Statistical Power Analyses for Windows was used to estimate the sample size [15].

The 1,000 independent older people were randomly selected, using the Basic Resident Register of the city from populations based on age group, i.e. 60–64, 65–69, 70–74, 75–79, 80–84, 85–89, 90–94 and ≥ 95 years, according to the proportion of each age group in the city’s population. In selecting dependent older people, we focused on long-term care insurance (LTCI) beneficiaries [16]. The Japanese Government started the national long-term care insurance (LTCI) system in 2000 based on the Long-Term Care Insurance Act [17]. Everyone aged ≥65 years, plus anyone aged 40–64 years with an aging-related disability (i.e., terminal cancer or rheumatoid arthritis), is eligible for LTCI. A care-needs level based on the total estimated care minutes is then assigned to each individual. The assessment is analyzed so that each applicant can be classified into one of seven levels (or rejected), according to the level of care needed [18] as shown in Table 1. The 2,000 LTCI beneficiaries living at home were randomly selected from among older individuals certified as requiring long-term care stratified by care level, according to the proportion of each care level, i.e., need support 1–2 and care levels 1–5. The Japanese version of the 10-Item Eating Assessment Tool (EAT-10) [14,19] was sent to participants by postal mail, and the returned questionnaires were used for the analysis. In order to improve the response rate, a public information magazine was used to attract older people’s attention to encourage them to return the questionnaire to the local government office. Furthermore, we held explanatory sessions to explain the importance of this survey to prevent pneumonia and maintain/improve health status for care support specialists in charge of the care of those certified as requiring long-term care in the community. The EAT-10 questionnaire was self-administered and the answers were recorded by the participants. If a participant without severe cognitive deficits, was unable to write responses to the items due to impaired upper limb function or was unable to read the items due to visual problems, his/her caregiver read out the items and wrote responses on behalf of the participant. In such cases, it was ensured that the caregiver did not respond to the items on his/her own thought. Participants with severe cognitive deficits or disturbance of consciousness, we explained his/her caregiver not to return. Only participants who responded to all items were included in the analysis and those with any missing responses were excluded.

**Table 1:** Long-term care insurance (LTCI) care needs levels of Japan.

Care level group	Eligibility criteria
Need support 1	> = 25,<32 estimated total care minutes per day)
Need support 2	> = 32, <50 estimated total care minutes per day) ^a^
Care Level 1	> = 32, <50 estimated total care minutes per day) ^a^
Care Level 2	> = 50, <70 estimated total care minutes per day)
Care Level 3	> = 70, <90 estimated total care minutes per day)
Care Level 4	> = 90, <110 estimated total care minutes per day)
Care Level 5	> = 110 estimated total care minutes per day)

^a:^ Level of stability is evaluated as clinical condition of causal diseases and dementia at the assessment conference

Prior to analysis, the independent older people were stratified into three groups by age: 65–74, 75–84, and ≥85 years. The LTCI beneficiaries were stratified into three groups by care level those in need levels 1/2 or care level 1 as the “low” care group, care levels 2/3 as the “moderate” care group, and care levels 4/5 as the “high” care group. The participants were also divided into two groups based on the EAT-10 score, with a score of ≥3 being defined as the cut-off value [13]. The percentage of participants with an EAT-10 score ≥3 was defined as the prevalence of suspected dysphagia [13,14]. The survey period was between November 20, 2015 and December 28, 2015.

### Ethical considerations

This study was conducted upon approval by the ethics committee at Nippon Dental University School of Life Dentistry (Approval No. NDU-T2015-46).

### Statistical analysis

SPSS Statistics Ver. 23 for Windows (IBM) was used. Chi-square test followed by residual analysis was used for analyzing the percentage and response rate of participants with and without suspected dysphagia. Analysis of the distribution of EAT-10 scores, and comparisons among items, age groups and care levels were performed using the Kruskal-Wallis test, and subsequent analysis was performed using the Mann-Whitney U test. Effect sizes were calculated using *Cramer’s V* for chi-square test and *r* for Mann-Whitney U test. Differences were considered statistically significant at alpha < 0.05. All values are presented as mean ± standard deviation (SD).

## Results

The number of participants and effective response rates in each group are shown in Tables 2 and 3. The effective response from the participants was sufficient enough for analysis.

**Table 2:** Effective response rate in independent older people.

Age group in years	Sending, n	ER, n (%)	*Cramer’s V*	*P*-Value^a^
Men				
65–74	195	119 (61.0)	0.126	0.063
75–84	122	89 (73.0)^b^		
≥85	33	19 (57.6)		
Total	350	227 (64.9)		
Women				
65–74	225	147 (65.3)^b^	0.142	0.009
75–84	154	95 (61.7)		
≥85	88	41 (46.6)^b^		
Total	467	283 (60.6)		
Total				
65–74	420	266 (63.3)	0.115	0.005
75–84	276	184 (66.7)		
≥85	121	60 (49.6)^b^		
Total	817	510 (62.4)		

The 10-Item Eating Assessment Tool (EAT-10) was send to 183 independent older people aged 60–64.

ER, Effective response

^a:^ chi-square test

^b:^ *P* < 0.05, residual analysis

**Table 3:** Effective response rate in long-term care insurance beneficiaries.

Care group	Sending, n	ER, n (%)	*Cramer’s V*	*P*-Value^a^
Men				
Low	350	197 (56.3)^b^	0.147	0.001
Moderate	212	97 (45.8)		
High	106	39 (36.8)^b^		
Total	668	333 (49.9)		
Women				
Low	696	337 (48.4)^b^	0.186	<0.001
Moderate	366	150 (41.0)		
High	2770	66 (2.4)^b^		
Total	1332	553 (41.5)		
Total				
Low	1046	534 (51.1)^b^	0.174	<0.001
Moderate	578	247 (42.7)		
High	376	105 (27.9)^b^		
Total	2000	886 (44.3)		

Low, need support 1/2 or care level 1; Moderate, care levels 2/3; High, care levels 4/5.

ER, Effective response

^a:^ chi-square test

^b:^ *P* < 0.05, residual analysis

### Independent older people

#### Eligible participants

The EAT-10 questionnaire was sent to 1,000 individuals. After excluding incomplete responses or who were 60–64 years old, 510 respondents (75.0 ± 7.2 years) were included in the survey. The effective response rates in the age groups 65–74, 75–84 and ≥85 were 63.3, 66.7 and 49.6%, respectively, showing a significant age-dependent difference (*P* = 0.005).

#### Prevalence of suspected dysphagia

The score of the EAT-10 is shown in Table 4. The mean EAT-10 score was 2.0 ± 3.6 and increased significantly with advancing age (*P* < 0.001). The percentage with EAT-10 scores ≥3 was 25.1% (128/510), and percentages differed significantly among age groups (*P* < 0.001).

**Table 4:** Eating Assessment Tool (EAT-10) scores in independent older people.

Age group in years	Mean ± SD	*Kruskal-Wallis H*	*P*-Value^a^	Score≥3, n (%)	*Cramer’s V*	*P*-Value^e^
Men						
65–74	1.3 ± 2.6^b^	11.359	0.003	21 (17.6)^f^	0.230	0.003
75–84	2.4 ± 4.1			26 (29.2)		
≥85	4.0 ± 4.5^d^			10 (52.6)^f^		
Total	1.9 ± 3.5			57 (25.1)		
Women						
65–74	1.8 ± 3.1	7.117	0.028	31 (21.1)	0.159	0.028
75–84	1.6 ± 2.7^c^			23 (24.2)		
≥85	3.7 ± 3.6^d^			17 (41.5)^f^		
65–74	2.0 ± 3.7			71 (25.1)		
Total						
65–74	1.6 ± 2.9	15.621	<0.001	52 (19.5)^f^	0.184	<0.001
75–84	2.0 ± 3.5^c^			49 (26.6)		
≥85	3.8 ± 5.8^d^			27 (45.0)^f^		
Total	2.0 ± 3.6			128 (25.1)		

SD: Standard deviation

^a:^ Kruskal-Wallis test

^b:^ *P* < 0.05 vs 75–84, Mann-Whitney U test

^c:^ *P* < 0.05 vs ≥85, Mann-Whitney U test

^d:^ *P* < 0.05 vs 65–74, Mann-Whitney U test

^e:^ chi-square test

^f:^ *P* < 0.05, residual analysis

effect size, *r;* Men, 65–74 vs. 75–84, -0.143; 65–74 vs. ≥85, -0.269; Women, 75–84 vs. 85, -0.215; 65–74 vs. ≥85, -0.176; Total, 75–84 vs. 85, -0.191; 75–84 vs. 85, -0.220

#### Analysis of score distribution

**Fig 1:**
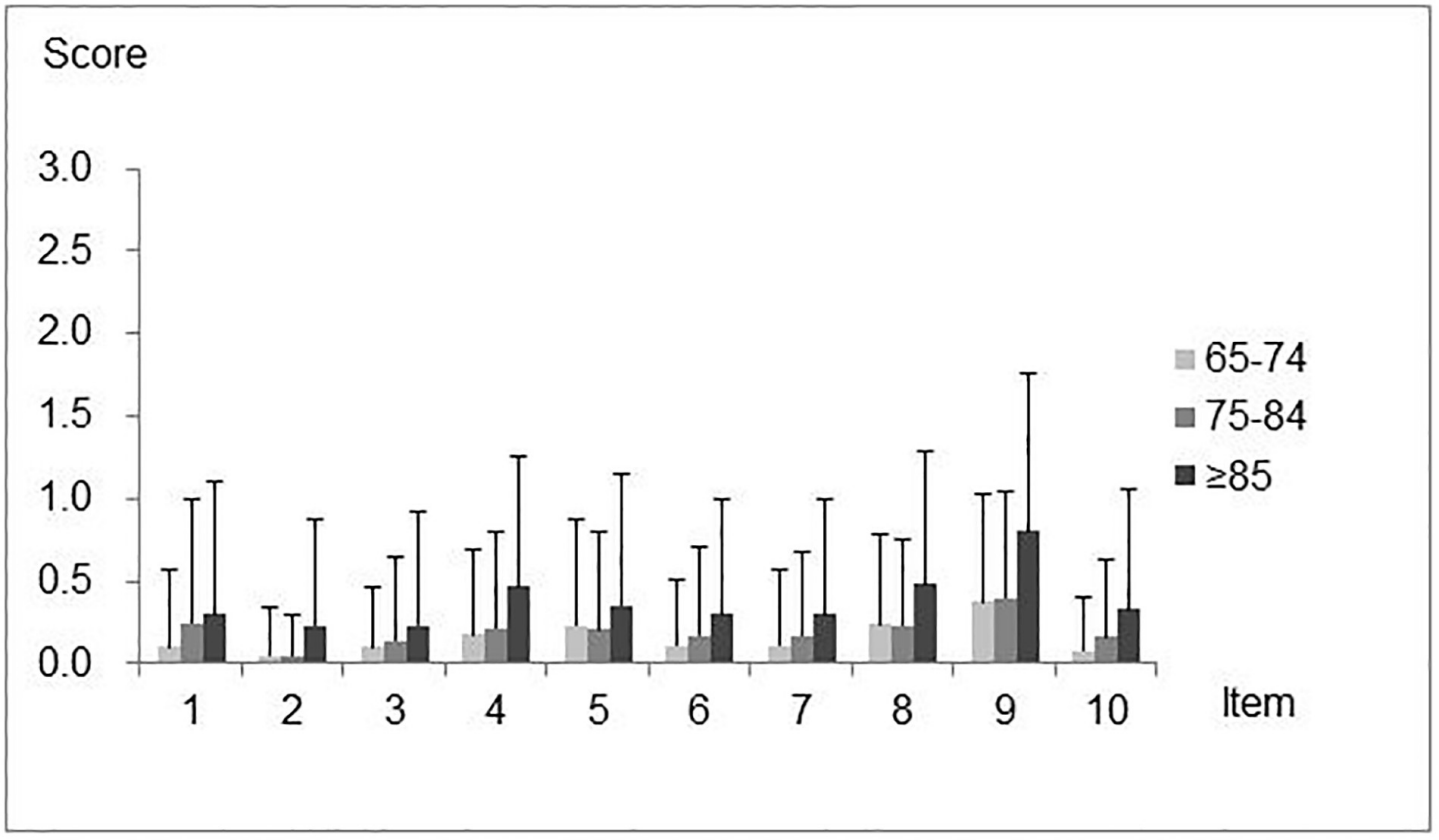
Mean Eating Assessment Tool (EAT-10) scores for each item in independent older people. Values are expressed as Mean and standard deviation (SD).

Distribution of scores is shown in Fig 1 and Table 5.

**Table 5:** Distribution of scores from three age groups relating to each item of the Eating Assessment Tool (EAT-10).

10-Item Eating Assessment Tool		Age group in years					
		65–74		75–84		≥85	
		Item^b^	Mean ± SD	Item^b^	Mean ± SD	Item^b^	Mean ± SD
1^a^	My swallowing problem has caused to lose weight.	4,5,8,9	0.10 ± 0.47^c^	2,9	0.25 ± 0.75	9	0.30 ± 0.81^e^
2^a^	My swallowing problem interferes with my ability to go out for meals.	3,4,5,6,8,9	0.05 ± 0.29	1,4,5,6,7,8,9,10	0.05 ± 0.25^d^	4,8,9	0.23 ± 0.65^e^
3	Swallowing liquids takes extra effort.	2,5,8,9	0.10 ± 0.37	8,9	0.14 ± 0.51	4,8,9	0.23 ± 0.70
4^a^	Swallowing solids takes extra effort.	1,2,6,	0.18 ± 0.51	2,9	0.22 ± 0.58^d^	2,3,9	0.47 ± 0.79^e^
		7,9,10					
5	Swallowing pills takes extra effort.	1,2,3,6,7,9,10	0.23 ± 0.64	2,9	0.21 ± 0.59	9	0.35 ± 0.80
6^a^	Swallowing is painful.	2,4,5,8,9	0.11 ± 0.39	2,9	0.17 ± 0.54	9	0.30 ± 0.70^e^
7^a^	The pleasure of eating is affected by my swallowing.	4,5,8,9	0.11 ± 0.47^c^	2,9	0.17 ± 0.51	9	0.30 ± 0.70^e^
8^a^	When I swallow food sticks in my throat.	1,2,3,4,5,9,10	0.24 ± 0.54	2,3,9	0.23 ± 0.53^d^	2,3,9	0.48 ± 0.81^e^
9^a^	I cough when I eat.	1,2,3,4,5,	0.38 ± 0.65	1,2,3,4,5,	0.40 ± 0.64^d^	1,2,3,4,5,	0.80 ± 0.95^e^
		6,7,8,10		6,7,8,10		6,7,8,10	
10^a^	Swallowing is stressful.	4,5,8,9	0.08 ± 0.33	2,9	0.16 ± 0.47^d^	9	0.33 ± 0.73^e^

SD: Standard deviation

^a:^ Statistical significance among age groups (*P* < 0.05, Kruskal-Wallis test)

^b:^ Item numbers with statistical significance in item comparisons (*P* < 0.05, Mann-Whitney U test)

^c:^ *P* < 0.05 vs 75–84, Mann-Whitney U test

^d:^ *P* < 0.05 vs ≥85, Mann-Whitney U test

^e:^ d: *P* < 0.05 vs 65–74, Mann-Whitney U test b: *P* < 0.05 vs 75–84, Mann-Whitney U test

*Score distribution for each item by age group*. In all age groups, item No. 9 “I cough when I eat.” was assigned significantly higher scores than the other items. In the 65–74 age group, item No. 4 “Swallowing solids takes extra effort.”, item No. 5 “Swallowing pills takes extra effort.” and item No. 8 “When I swallow food sticks in my throat.” were also assigned higher scores, while item No. 2 “My swallowing problem interferes with my ability to go out for meals.” was assigned a significantly lower score. In the 75–84 age group, item No. 2 was assigned a significantly lower score than the other items. In the ≥85 age group, the distribution of scores differed less among items, although item No. 9 was assigned the highest score.

*Score distribution for age groups by each item*. In all age groups, significant differences were observed for all items except for item No. 3 “Swallowing liquids takes extra effort.” and item No. 5. Only two items showed significant differences between the 65–74 and 75–84 age groups. Significant differences between the 75–84 and ≥85 age groups were observed for five items. Significant differences were observed for all items except for No. 3 and No. 5 between the ≥85 and 65–74 age groups.

### Long-term care insurance beneficiaries

#### Eligible participants

The EAT-10 questionnaire was sent to 2,000 individuals. After excluding incomplete responses, 886 respondents (mean age: 82.3 ± 6.7 years) were included. The effective response rates in the low, moderate and high care level groups were 51.1, 42.7, and 27.9%, respectively, showing significant differences depending on care levels (*P* < 0.001).

#### Prevalence of suspected dysphagia

The score of the EAT-10 is shown in Table 6. The mean EAT-10 score was 5.8 ± 6.9 and increased significantly as the care level increased (*P* < 0.001). The percentage of participants with EAT-10 scores ≥3 was 53.8% (477/886), and percentages differed significantly among care level groups (*P* < 0.001).

**Table 6:** Eating Assessment Tool (EAT-10) scores in long-term care insurance beneficiaries.

Care group	Mean ± SD	*Kruskal-Wallis H*	*P*-Value^a^	Score≥3, n (%)	*Cramer’s V*	*P*-Value^e^
Men						
Low	4.9 ± 6.5	14.160	0.001	99 (50.3)	0.119	0.094
Moderate	5.7 ± 6.1^c^			51 (52.6)		
High	10.2 ± 10.0^d^			27 (69.2)^f^		
Total	5.8 ± 7.1			177 (53.2)		
Women						
Low	4.7 ± 5.9^b^	25.778	<0.001	167 (49.6)^f^	0.142	0.004
Moderate	6.3 ± 6.8^c^			86 (57.3)		
High	10.4 ± 8.9^f^			47 (71.2)^f^		
Total	5.8 ± 6.8			300 (54.2)		
Total						
Low	4.8 ± 6.1^b^	39.745	<0.001	266 (49.8)^f^	0.132	<0.001
Moderate	6.1 ± 6.5^c^			137 (55.5)		
High	10.4 ± 9.3^d^			74 (70.5)^f^		
Total	5.8 ± 6.9			477 (53.8)		

SD: Standard deviation

^a:^ Kruskal-Wallis test

^b:^ *P* < 0.05 vs moderate, Mann-Whitney U test

^c:^ *P* < 0.05 vs High, Mann-Whitney U test

^d:^ *P* < 0.05 vs Low, Mann-Whitney U test

^e:^ chi-square test

^f:^ *P* < 0.05, residual analysis

effect size, *r;* Men, Moderate vs. High, -0.220; Low vs. High, -0.236; Women, Low vs Moderate, -0.105; Moderate vs. High, -0.212; Low vs High, -0.243; Total, Low vs Moderate, -0.105; Moderate vs. High, -0.213; Low vs High, -0.240

#### Analysis of score distribution

**Fig 2:**
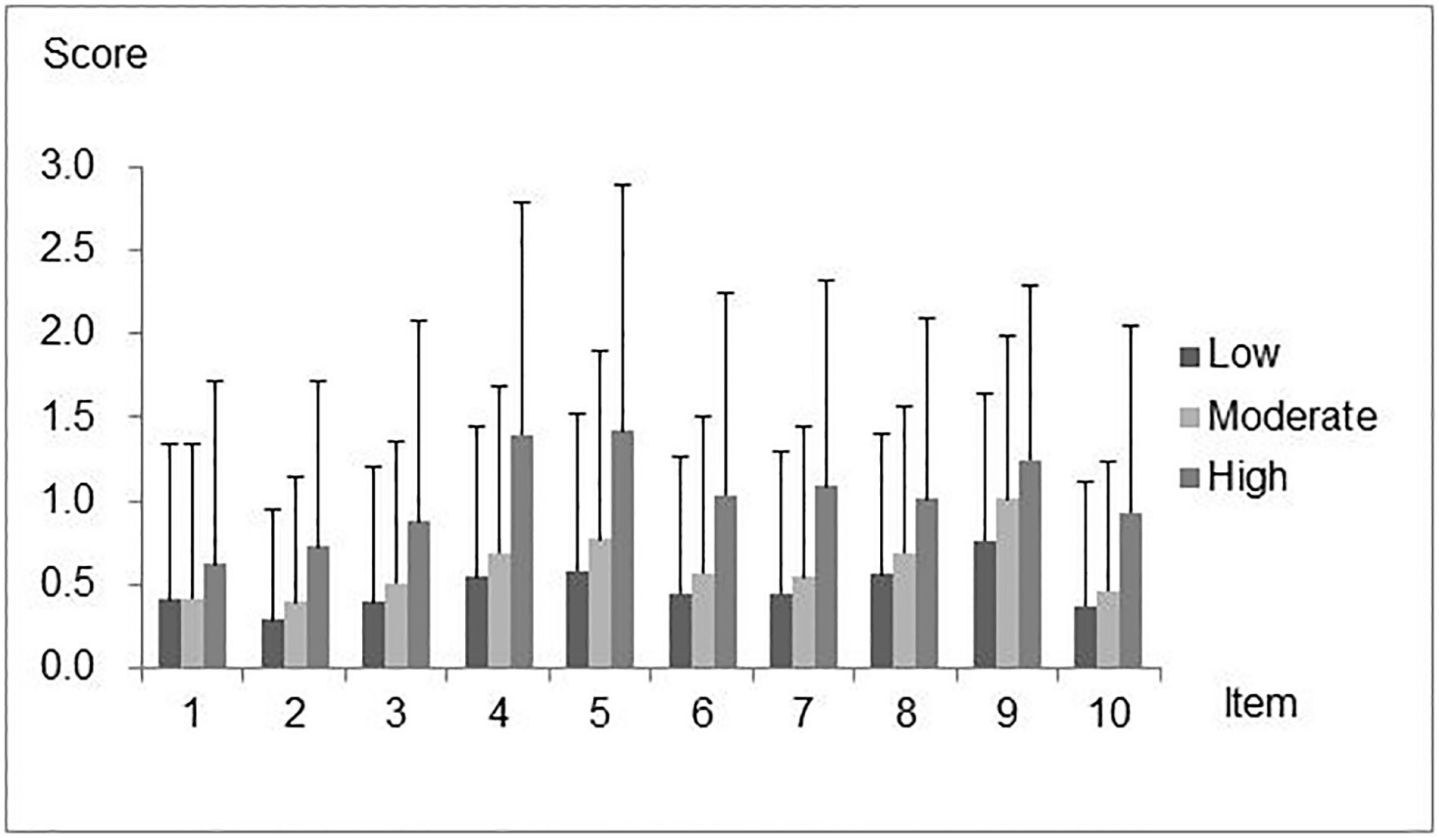
Mean Eating Assessment Tool (EAT-10) scores for each item in independent older people in long-term care insurance beneficiaries. Values are expressed as Mean and standard deviation (SD).

Distribution of scores is shown in Fig 2 and Table 7.

**Table 7:** Distribution of scores from three care groups of participants relating to each item of the Eating Assessment Tool (EAT-10).

10-Item Eating Assessment Tool		Care group					
		Low		Moderate		High	
		Item^b^	Mean ± SD	Item^b^	Mean ± SD	Item^b^	Mean ± SD
1^a^	My swallowing problem has caused to lose weight.	4,5,6,7,8,9	0.41 ± 0.93	2,3,4,5,6,7,8,9,10	0.42 ± 0.92^d^	4,5,6,7,8,9,10	0.62 ± 1.10
2^a^	My swallowing problem interferes with my ability to go out for meals.	3,4,5,6,7,8,9,10	0.29 ± 0.67^c^	4,5,6,7,8,9	0.39 ± 0.75^d^	4,5,7,8,9	0.72 ± 1.00
3^a^	Swallowing liquids takes extra effort.	2,4,5,8,9	0.40 ± 0.81^c^	1,4,5,8,9	0.51 ± 0.84^d^	4,5,9	0.88 ± 1.21
4^a^	Swallowing solids takes extra effort.	1,2,3,	054 ± 0.90^c^	1,2,3,	0.69 ± 0.99^d^	1,2,3,10	1.40 ± 1.38^e^
		7,9,10		9,10			
5^a^	Swallowing pills takes extra effort.	1,2,3,6,7,9,10	0.59 ± 0.94^c^	1,2,3,7,9,10	0.77 ± 1.13^d^	1,2,3,10	1.42 ± 1.47^e^
6^a^	Swallowing is painful.	1,2,5,8,9	0.45 ± 0.81	1,5,8,9	0.57 ± 0.93^d^	1	1.04 ± 1.22^e^
7^a^	The pleasure of eating is affected by my swallowing.	1,2,4,5,8,9	0.45 ± 0.85	1,5,8,9	0.54 ± 0.91^d^	1,2	1.09 ± 1.24^e^
8^a^	When I swallow food sticks in my throat.	1,2,3,6,7,9,10	0.56 ± 0.84^c^	1,2,3,6,7,9,10	0.69 ± 0.88^d^	1,2	1.01 ± 1.09^e^
9^a^	I cough when I eat.	1,2,3,4,5,6,7,8,10	0.77 ± 0.88^c^	1,2,3,4,5,6,7,8,10	1.01 ± 0.99	1,2,3,10	1.25 ± 1.05^d^
10^a^	Swallowing is stressful.	2,4,5,8,9	0.37 ± 0.75^c^	1,4,5,8,9	0.47 ± 0.77^d^	1,4,5,9	0.93 ± 1.12^e^

SD: Standard deviation

^a:^ Statistical significance among care groups (*P* < 0.05, Kruskal-Wallis test)

^b:^ Item numbers with statistical significance in item comparisons (*P* < 0.05, Mann-Whitney U test)

^c:^ *P* < 0.05 vs moderate, Mann-Whitney U test

^d:^ *P* < 0.05 vs High, Mann-Whitney U test

^e:^ *P* < 0.05 vs Low, Mann-Whitney U test

*Score distribution for each item by care level*. In the low and moderate care level groups, item No. 9 was assigned significantly higher scores than the other items. Item No. 8 was also assigned higher scores than many other items. In contrast, item No. 2 and item No. 1 “My swallowing problem has caused me to lose weight.” were assigned significantly lower scores in the low and moderate care level groups. In the high care level group, apart from item No. 9, items No. 4, 5, 8, 6 “Swallowing is painful.” and 7 “The pleasure of eating is affected by my swallowing” were also assigned high scores, whereas item No. 1 was assigned a low score.

*Score distribution for care levels by each item*. In all care level groups, significant differences were observed for all items. Seven items showed significant differences between the low and moderate care level groups. Significant differences were observed for all items, except for item No. 9, between the moderate and high care level groups. Significant differences were observed for all items between the low and high care level groups.

## Discussion

In this study, the prevalences of suspected dysphagia in independent and dependent older people were 25. 1 and 53.8%, respectively, demonstrating a substantial proportion of individuals in both groups to have suspected dysphagia. We found interesting features: the distribution of scores for the questionnaire items differed with increase in age and care level, and older people became aware of more subjective symptoms with increase in age and care level. Coughing was found to be the easiest item to notice, and people with higher care levels were more likely to experience difficulties in swallowing solids and to experience a mental burden. These results could contribute to promotion of eating/swallowing support in the community.

The EAT-10 was developed by Belafsky *et al*. in 2008 [13]. It consists of 10 items and each item is answered on a 5-point scale from 0 to 4, and in individuals with more than 3-points, dysphagia is suspected and referral to a specialist is recommended. Translated versions of the questionnaire are currently available in various countries with demonstrated reliability and validity, and its usefulness has come to be widely accepted. Several investigations have focused on the prevalence of dysphagia in independent older people using questionnaires, interviews and simple tests. According to the results obtained, the prevalence of dysphagia was 13.8% in a study reported by Kawashima *et al*., using the Dysphagia Screening Questionnaire [20], 32.5% in an interview survey using the MDADI reported Nelson *et al* [21] and 27.2% in a study reported by Serra-Prat *et al*. using a Volume-Viscosity Swallow Test (V-VST) [12]. Among these, the study conducted by Serra-Prat *et al*. [12] was the only one involving participants selected by stratified randomization. In a survey of Japanese LTCI beneficiaries, the prevalence was 35.3% in participants requiring a relatively low level of care as selected by 3-oz water test and a questionnaire; dysphagia risk assessment for the community-dwelling elderly (DRACE), as reported by Miura *et al* [22]. In a survey involving nursing home residents, using the Gugging Swallowing Screen (GUSS) test, the prevalence was reported to be 52.7% [23]. Even higher prevalences have been reported in studies targeting different hospitals and participants [24]. As such, most of the previous studies investigating the prevalence of dysphagia included only specific populations of participants. Therefore, we used the Basic Resident Register and long-term care insurance information to stratify and randomly select participants in order to avoid biased selection.

In independent older people, the prevalence increased significantly with advancing age. The increasing prevalence of dysphagia with age is consistent with previous reports [25]. The fact that similar results were obtained from the independent older people suggests age-related physiological changes to impact eating/swallowing functions. The prevalence increased significantly as the long-term care level rose. The rising prevalence of dysphagia with increasing care level is consistent with previous reports [26]. It is thus likely that older people with decreased activities of daily living and cognitive function, and certified as requiring long-term care have impaired eating/swallowing functions, as well as impaired physical functions. Around 20% of individuals certified as requiring long-term care have late effects of cerebrovascular disease [27, 28]. Cerebrovascular disease is reportedly a common cause of dysphagia, with an increasing severity of late effects being associated with an increasing severity of dysphagia [28]. Many of the individuals certified as requiring high-level care are known to have neuromuscular diseases, which are also associated with severe dysphagia [29]. This may explain the high prevalence of dysphagia in the high care level group in the present study.

In independent older people, coughing was the most perceivable symptom. Many self-administered and question-based screening methods include questions about choking/coughing while eating [20–22,30]. It is inferred that this item is an important question to estimate the presence of dysphagia. Questions about swallowing of solids were also assigned high perceivable symptom, indicating that questions regarding difficulty when swallowing solids are effective for estimating the presence of dysphagia [20–22,30]. However, the differences between scores for these swallowing solid-related complaints and those for other questions decreased. This was probably due to uniformly rising incidences of other dysphagia symptoms/signs with advancing age.

In dependent older people, question about ability to go out for meals was assigned lower perceivable symptom than the other items. A possible explanation is that since aging is associated with a decreased frequency of going out [31], older people who do not go out often are less likely to be prevented from going out by dysphagia. In the comparisons among age groups of the independent older people, significant differences were observed among the three groups for all items except for questions about swallowing liquid and pills. This suggested that these problems are considered to be easily overcome by way of drinking water [32] and the use of easy-to-take dosage forms [33]. In dependent older people, coughing was the most perceivable symptom, as well as in independent older people. Apart from coughing, an increased care level was associated with an increased frequency of problems with swallowing of solids and psychological burden in high care level. Dependent older people are more likely to have lower physical functions. Declining physical functioning is associated with both whole-body and swallowing-related muscle strength [34,35]. Since swallowing power is necessary for a solid diet, those with deteriorated physical functioning may have a problem swallowing solid food. In addition, those with people high care levels have a high proportion of various symptoms. Therefore, individuals requiring high-level care may have a greater psychological burden accompanying difficulty in swallowing solids. The question about weight loss was also assigned a low perceivable symptom. In older people, gradual weight loss occurs without their noticing and it tends to be overlooked [36]. In a study reported by Izawa *et al*., 292 of 952 (30.7%) older individuals receiving care had no available data on body weight and did not know their own weight [37]. These observations suggest that body weight is difficult to measure in older individuals requiring long-term care and even if it is measured, possibly in a clinical setting, they may not know their own weight. It is thus likely that dependent participants tended not to recognize weight loss as describing a subjective symptom. There were significant differences among all care level groups, showing that rising care level was associated with an increasing degree of perceivable symptom.

This survey was carried out as part of a regional survey on eating/swallowing, conducted by a local government. The population of this city was approximately 180,000. In Japan, about 70% of people live in cities with populations of more than 100,000 [38]. Therefore, it can be said that this city is a standard city in Japan, and the results of this study provide valuable data for understanding the current swallowing problems in older people living in the community. Based on these results, a primary support plan to prevent pneumonia has already been promoted in this city.

This research has limitations. First, despite our efforts, the response rates were low in independent older people aged 85 years or older and in people with high care levels. This may have influenced the results. Second, the lack of data on medical history and physical/cognitive functioning might have been a source of bias. Third, we only used the EAT-10, and objective examinations were not performed to diagnose dysphagia. These seem to be limitations of the EAT-10, as it is a self-administered questionnaire for dysphagia screening.

### Conclusion

In independent people, approximately one in four individuals showed suspected dysphagia and coughing was the most perceivable symptom. In dependent people, approximately one in two individuals showed suspected dysphagia and their specifically perceivable symptoms were coughing, difficulties in swallowing solids and psychological burden.

## Supporting information

S1 File data set. (XLSX)
